# Gradual Changes of Gut Microbiota in Weaned Miniature Piglets

**DOI:** 10.3389/fmicb.2016.01727

**Published:** 2016-11-02

**Authors:** Jun Hu, Yangfan Nie, Jianwei Chen, Yong Zhang, Zhichang Wang, Qiwen Fan, Xianghua Yan

**Affiliations:** ^1^Department of Animal Nutrition and Feed Science, College of Animal Sciences and Technology, Huazhong Agricultural UniversityWuhan, China; ^2^The Cooperative Innovation Center for Sustainable Pig ProductionWuhan, China; ^3^Institute of Marine Omics Research, Beijing Genomics Institute–ShenzhenShenzhen, China; ^4^Key Laboratory of Animal Genetics, Breeding and Reproduction in the Plateau Mountainous Region, Ministry of Education/Guizhou UniversityGuiyang, China

**Keywords:** Gut microbiota, Congjiang miniature piglet, *Lactobacillus coleohominis*, *Eubacterium hallii*, PICRUSt

## Abstract

Colonization of gut microbiota in mammals during the early life is vital to host health. The miniature piglet has recently been considered as an optimal infant model. However, less is known about the development of gut microbiota in miniature piglets. Here, this study was conducted to explore how the gut microbiota develops in weaned Congjiang miniature piglets. In contrast to the relatively stabilized gut fungal community, gut bacterial community showed a marked drop in alpha diversity, accompanied by significant alterations in taxonomic compositions. The relative abundances of 24 bacterial genera significantly declined, whereas the relative abundances of 7 bacterial genera (*Fibrobacter, Collinsella, Roseburia, Prevotella, Dorea, Howardella*, and *Blautia*) significantly increased with the age of weaned piglets. Fungal taxonomic analysis showed that the relative abundances of two genera (*Kazachstania* and *Aureobasidium*) significantly decreased, whereas the relative abundances of four genera (*Aspergillus, Cladosporium, Simplicillium*, and *Candida*) significantly increased as the piglets aged. *Kazachstania telluris* was the signature species predominated in gut fungal communities of weaned miniature piglets. The functional maturation of the gut bacterial community was characterized by the significantly increased digestive system, glycan biosynthesis and metabolism, and vitamin B biosynthesis as the piglets aged. These findings suggest that marked gut microbial changes in Congjiang miniature piglets may contribute to understand the potential gut microbiota development of weaned infants.

## Introduction

The mammalian intestine harbors trillions of microbes which play vital roles in nutrient absorption and metabolism (Backhed et al., 2007), the host immune defense system development (Ivanov et al., 2009), the intestinal epithelium differentiation (Sommer and Bäckhed, 2013), and intestinal mucosal barrier maintenance (Garrett et al., 2010). In recent years, studies on the development of gut microbiota have absorbed a mass of attentions (Backhed et al., 2015; Kostic et al., 2015). The colonization of infant intestinal microbiota begins *in utero* (Aagaard et al., 2014) and is influenced by the diet and other environmental factors (Eggesbo et al., 2011; Koenig et al., 2011; La Rosa et al., 2014). The initial development of gut microbiota has long-term physiological influences on the host (Foxx-Orenstein and Chey, 2012).

There has been a great interest in the studies on the gut microbiota using pigs as models due to their similarities to human beings in relation to anatomy and nutritional physiology (Garthoff et al., 2002; Pang et al., 2007; Heinritz et al., 2013; Kim and Isaacson, 2015). Although much studies have done on the development of gut microbiota in adult pigs and its relationship with antibiotics treatment, few studies have been focused on the development of gut microbiota in piglets (Kim et al., 2012; Looft et al., 2012). Weaning is an inevitable and important event for infants and piglets, whereas may cause intestinal microflora-related disorders, such as diarrhea (Smith et al., 2010; Fawzy et al., 2011). Moreover, miniature piglets have physiological and anatomic similarities to human beings, especially in infancy (Shulman et al., 1988; Garthoff et al., 2002; Vodicka et al., 2005). Thus, the development of gut microbiota in miniature weaned piglets is of great significance. Previous studies on the gut microbiota in pigs were based on the bacterial communities and extremely few studies have explored the fungal communities in pigs (Kim and Isaacson, 2015). However, growing evidences have revealed the important relationships between gut fungal communities and the host health (Liggenstoffer et al., 2010; Iliev et al., 2012). So the characterizations of fungal communities in pigs require further investigation.

The present study was focused on the development of gut bacterial and fungal communities in Congjiang miniature piglets, a Chinese native pig breed, during the early period after weaning. The gut bacterial and fungal communities in weaned piglets were characterized by 16S ribosomal DNA (16S rDNA) and Internal Transcribed Spacer 2 (ITS2) high-throughput sequencing, two culture-independent methods, respectively. The functional profiles of gut bacterial communities in weaned piglets were analyzed using Phylogenetic investigation of communities by reconstruction of unobserved states (PICRUSt). This study provided an insight into the shifts in gut microbial diversity, taxonomic composition, and functional profile of Congjiang miniature piglets during the early period after weaning.

## Materials and methods

### Animals and sample collection

A total of 30 Congjiang miniature piglets, with similar body weight at the age of 21 days, were used in this study. Piglets were weaned at the age of 21 days and randomly split into 3 pens. Each pen contained 10 piglets and all the piglets had free access to diets and water. One piglet was randomly selected from each pen. A total of 3 piglets selected were ear tagged for identification. Fresh feces were individually collected from the ear-tagged piglets at 3, 5, 6, 8, and 11 days after weaning. To obtain representative fecal samples from each piglet, we firstly collected the fresh feces from one piglet as much as possible and then mixed well the feces immediately. A total 15 fresh feces samples individually collected were frozen in liquid nitrogen immediately and then stored at −80°C before microbial genomic DNA extraction. Piglets handling protocols (permit number: HZAUSW2013-0006) were approved by the Institutional Animal Care and Use Committee of Huazhong Agricultural University. The methods were carried out in accordance with the approved guidelines.

### Microbial genomic DNA extraction

Total microbial genomic DNA, including bacterial and fungal genomic DNA in the feces of piglets, was extracted using a combined method of cetyl trimethyl ammonium bromide (CTAB) and bead-beating. Briefly, 0.25–0.30 g frozen feces were re-suspended in 1.5 ml ice-cold PBS and then were centrifuged at 9000 rpm for 10 min at 4°C to obtain microbial pellets. The pellets were washed in ice-cold PBS repeatedly until the supernatant became clear. Subsequently, the microbial pellets were re-suspended in 800 μl CTAB buffer containing 50 mM CTAB, 1.4 M NaCl, 100 mM Tris-HCl, 20 mM Ethylene Diamine Tetraacetic Acid (EDTA) and then were lysed by beat-beading using FastPrep-24 bead beater (MP Bio) at the top speed for total 240 s with an ice-cold bath for 120 s at the interval. After incubation at 70°C for 20 min, homogenate solution was centrifuged at 10,000 rpm for 10 min to obtain the supernatant. Five microliter of RNAase (10 mg/ml) was added into the supernatant obtained and the solution was incubated at 37°C for 30 min to remove the RNA. After that, three rounds of phenol: chloroform: isoamyl alcohol (V/V/V = 25: 24: 1) extraction were performed. The microbial DNA obtained was precipitated with the solution containing 1.5 ml ice-cold 95% ethanol and 40 μl 3M NaAc (20:1) at −20°C overnight and then re-suspended in 50 ml of Tris-EDTA buffer. Microbial genomic DNA was quantified using Qubit® 3.0 Fluorometer (Life technology) and DNA integrity was determined by gel electrophoresis (concentration of agarose gel: 1%, voltage: 150 V, and electrophoresis time: 40 min). Finally, the DNA samples examined were stored at −80°C until processing.

### 16S rDNA and ITS genes amplification and high-throughput sequencing

V4 region of bacterial 16S rDNA gene and ITS2 region of fungal ITS gene were amplified to construct DNA libraries for sequencing, respectively. Below were the key steps for V4 and ITS2 regions amplification and sequencing using an Illumina MiSeq platform. After genomic DNA concentration and integrity testing, 30 ng genomic DNA was used to run Polymerase Chain Reaction (PCR) per reaction. Briefly, dual-index fusion PCR primer cocktail, PCR master mix, and 30 ng genomic DNA were mixed to run the 50-ml V4 region PCR reactions. The primer sequences for V4 region amplification were 5′–NNNNNNNNGTGTGCC AGCMGCCGCGGTAA–3′ (forward) and 5′–GGACTACHV GGGTWTCTAAT–3′ (reverse). The melting temperature was 56°C and PCR cycle was 30. The primer sequences for ITS2 region amplification were 5′–NNNNNNNNGCATCGA TGAAGAACGCAGC–3′ (forward) and 5′–TCCTCCGCT TATTGATATGC–3′ (reverse). The melting temperature was 58°C and PCR cycle was 35. The PCR primer barcodes contributed to the segregation of sequencing information output based on the sampling numbers. All the PCR products were purified with AMPure XP beads (AGENCOURT) to remove the unspecific products. The final DNA libraries were validated in following ways: the average molecule length of amplifications were determined using the Agilent 2100 bioanalyzer instrument (Agilent DNA 1000 Reagents) and the DNA libraries were quantified by real-time quantitative PCR (qPCR) (EvaGreen™). Finally, the validated libraries were sequenced pair end on the Illumina Miseq system with the sequencing strategy PE250 (PE251 + 8 + 8 + 251) (Miseq reagent kit).

### Sequencing data analysis

In order to obtain more accurate and reliable results in subsequent bioinformatics analysis, the raw data from Illumina Miseq high-throughput sequencing will be pre-processed to eliminate the adapter pollution and low quality for obtaining clean reads by the following procedures: (1) those sequence reads not having an average quality of 30 over a 25 bp sliding window based on the phred algorithm were truncated and those trimmed reads having < 60% of their original length, as well as its paired read, were also removed; (2) those reads contaminated by adapter (default parameter: 15 bases overlapped by reads and adapter with maximal 3 bases mismatch allowed) were removed; (3) those reads with ambiguous base (N base), and its paired reads were removed; (4) those reads with low complexity (default: reads with 10 consecutive same base) were removed. The paired-end clean reads with overlap were merged to tags using Connecting Overlapped Pair-End (COPE, V1.2.1) (Liu et al., 2012) software. Subsequently, bacterial tags were clustered into Operational Taxonomic Units (OTUs) at 97% sequence similarity by scripts of Mothur (v1.31.2) (Schloss et al., 2009) software. Bacterial OTU representative sequences were taxonomically classified by scripts of Mothur (v1.31.2) software based on the Ribosomal Database Project (RDP) database (Cole et al., 2009). Fungal tags were clustered into OTUs at 97% sequence similarity by scripts of USEARCH (v7.0.1090) (Edgar, 2013) software. Fungal OTU representative sequences were taxonomically classified using RDP Classifier v.2.2 based on the UNITE database (Abarenkov et al., 2010). Venn diagram, which visually displays the numbers of common and unique OTUs among groups, was drawn by the package “VennDiagram” of R (v3.0.3) software. Principal component analysis (PCA) based on OTUs abundance was drawn by the package “ade4” of R (v3.0.3) software. Genus-level phylogenetic tree was constructed using the Quantitative Insights Into Microbial Ecology (QIIME) (v1.80) (Caporaso et al., 2010) built-in scripts and was imaged by R (v3.0.3) software at last. Chao index, Shannon index, and Simpson index which reflect alpha diversity were calculated by Mothur (v1.31.2) and the corresponding rarefaction curve are drawn by R (v3.0.3) software. Beta diversity based on weighted UniFrac distance was performed by QIIME (v1.80) software and displayed by the principal coordinates analysis (PCoA). Heat maps were generated using the package “gplots” of R (v3.0.3) software. The distance algorithm was “Euclidean” and the clustering method was “complete.”

### Functional profiles analysis of bacterial community using PICRUSt

16S rDNA gene studies were frequently performed to identify the bacterial taxonomic composition of environmental samples, but cannot be directly used to identify the functional capabilities of the bacteria. Here, PICRUSt method was applied for predicting the gene family abundances of bacterial communities based on the 16S rDNA gene data and a database of reference genomes (Langille et al., 2013). Briefly, the PICRUSt which consisted of two steps: gene content inference and metagenome inference was performed as described previously (Langille et al., 2013). In addition, the prediction accuracy of PICRUSt was evaluated by the Nearest Sequenced Taxon Index (NSTI), with lower value indicating a higher accuracy of prediction.

### Statistical analysis

Statistical analyses were carried out using GraphPad Prism (version 6.0c) software, R (v3.0.3) software, Metastats (White et al., 2009), and STAMP (Statistical Analysis of Metagenomic Profiles; Parks et al., 2014). Statistical comparisons of weighted UniFrac distances among groups were performed by the analysis of similarities (ANOSIM). The ANOSIM was conducted using the package “vegan” of R (v3.0.3) software. One-way analysis of variance (ANOVA) with Bonferroni's multiple comparison test was used for the comparison of alpha diversities among groups. Metastats software was used to identify the differentially abundant taxa (phyla, genera, and species) among groups. After the statistical comparison of taxa, we used the Benjamini-Hochberg to control the false discovery rate using the package “p. adjust” of R (v3.0.3) software. STAMP software was applied to detect the differentially abundant Kyoto Encyclopedia of Genes and Genomes (KEGG) pathways among groups with false discovery rate correction. *P*-value (corrected) < 0.05 was considered to indicate statistical significance.

## Results

### 16S rDNA and ITS sequence data from the gut microbiota in weaned piglets

**Figure 1:**
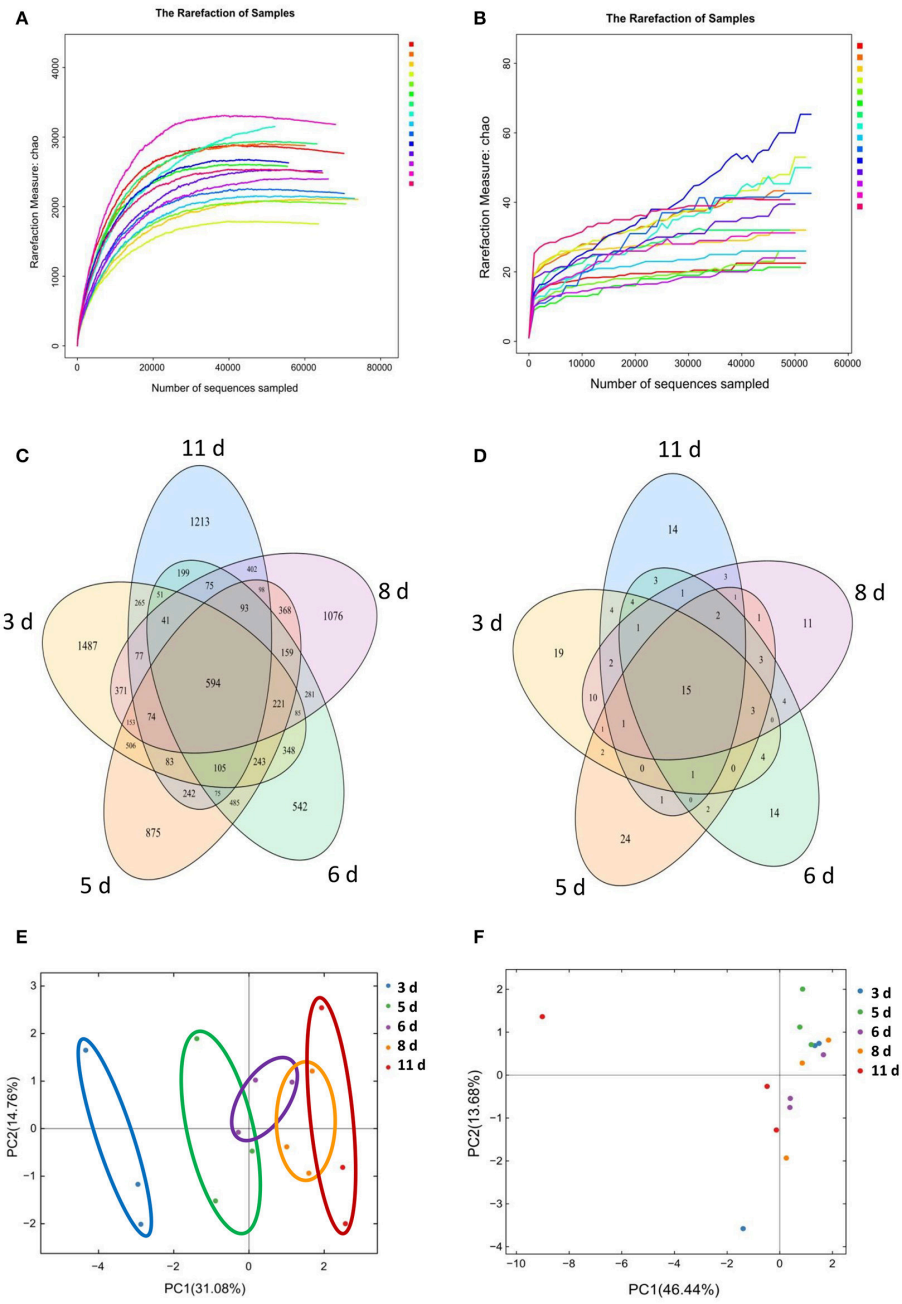
**Gut microbial OTUs development as the miniature piglets aged. (A)** Bacterial rarefaction curves based on Chao index were used to assess the depth of coverage for each sample. Each sample was distinguished by different colors of lines. **(B)** Fungal rarefaction curves based on Chao index were used to assess the depth of coverage for each sample. Each sample was distinguished by different colors of lines. **(C)** Venn diagrams for bacterial OTUs compositions (3 d, 3 days after weaning; 5 d, 5 days after weaning; 6 d, 6 days after weaning; 8 d, 8 days after weaning; 11 d, 11 days after weaning). **(D)** Venn diagrams for fungal OTUs compositions. **(E)** Scatterplot from PCA of bacterial OTUs in each sample. **(F)** Scatterplot from PCA of fungal OTUs in each sample.

To investigate the gut microbiota development in the Congjiang miniature piglets, this study amplicon-sequenced fecal samples from the weaned piglets at 5 sampled time points (3, 5, 6, 8, and 11 days) after weaning (Figure S1). We totally collected 973,050 and 769,660 high-quality sequences of V4 region and ITS2 region in 15 fecal samples from piglets after quality control, respectively. The average numbers of high-quality sequences generated per sample were 64,870 and 51,310 from bacterial and fungal populations, respectively. Rarefaction curves demonstrated that almost all the bacterial and fungal species were detected in feces of weaned piglets (Figures 1A,B).

Based on 97% sequence similarity, all the sequences of V4 region and ITS2 region were clustered into 10,887 bacterial OTUs and 151 fungal OTUs, respectively. There were 594 core OTUs in bacterial communities and 15 core OTUs in fungal populations, respectively (Figures 1C,D). PCA based on the bacterial OTUs showed that the samples clustered together according to age, and indicated a shift in the gut bacterial community with the age of piglets (Figure 1E). However, PCA based on the fungal OTUs demonstrated the samples cannot cluster together according to the age of piglets (Figure 1F).

### Shifts in gut microbial diversities with the age of weaned piglets

**Figure 2:**
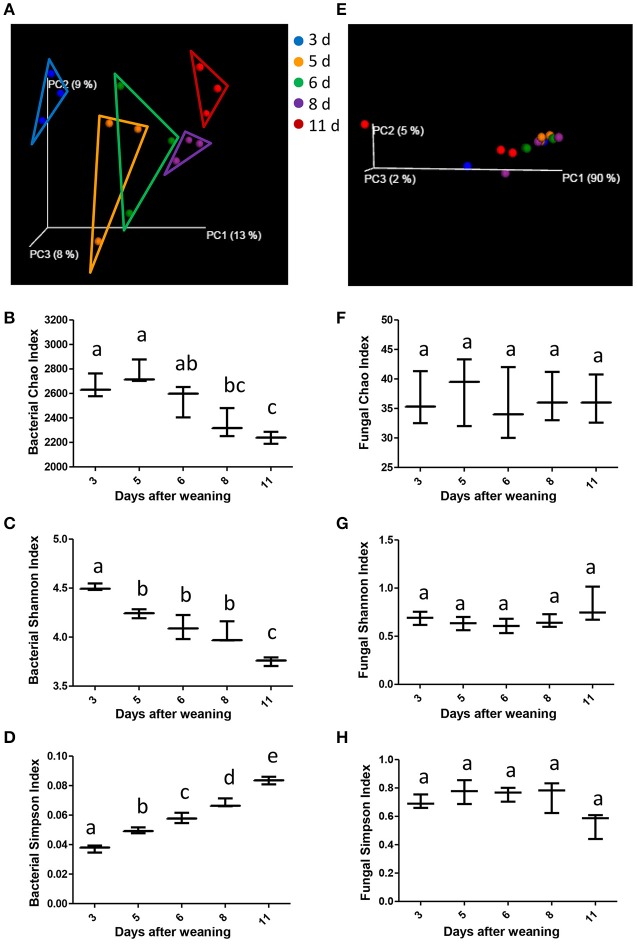
**The changes of gut microbial diversities as the miniature piglets aged. (A)** Scatterplot from PCoA, based on weighted UniFrac distance in bacterial communities (3 d, 3 days after weaning; 5 d, 5 days after weaning; 6 d, 6 days after weaning; 8 d, 8 days after weaning; 11 d, 11 days after weaning). **(B)** Bacterial alpha diversity determined by Chao index. **(C)** Bacterial alpha diversity determined by Shannon index. **(D)** Bacterial alpha diversity determined by Simpson index. **(E)** Scatterplot from PCoA, based on weighted UniFrac distance in fungal communities. **(F)** Fungal alpha diversity determined by Chao index. **(G)** Fungal alpha diversity determined by Shannon index. **(H)** Fungal alpha diversity determined by Simpson index. Different letters above the bars denotes a significantly different alpha diversity index among groups.

Using weighted UniFrac distances to evaluate the beta diversity (that is, diversity between individuals), the present study revealed that despite of shared environment and diets, the miniature piglets showed continuous alterations in their gut bacterial communities with age shown in the scatterplot from PCoA (Figure 2A). ANOSIM of weighted UniFrac distances indicated that significant separation of gut bacterial community by the age of miniature piglets (*R* = 0.6015, *P* = 0.001). To further dissect the dynamics of gut bacterial communities during the early period after weaning, we evaluated the alpha diversity in bacterial communities. The results indicated that Chao index which reflects the species richness significantly decreased with age (Figure 2B). Shannon index, which reflects the species richness and evenness, significantly decreased with age (Figure 2C). Simpson index, which also reflects the species richness and evenness, significantly increased with age (Figure 2D). Thus, the weaned piglets showed continuously decreased alpha diversities in their gut bacterial communities with age during the early period after weaning. In contrast, the samples didn't cluster together according to age, shown in the scatterplot from PCoA based on the fungal communities (Figure 2E). ANOSIM of weighted UniFrac distances demonstrated that there was no significant difference in the bacterial communities among groups (*R* = 0.0489, *P* = 0.303). Furthermore, there is also no significant alteration in the gut fungal alpha diversity with age, suggesting the relatively stabilized gut fungal communities in weaned piglets during the early period after weaning (Figures 2F–H).

### Significant alterations in the gut bacterial taxonomic compositions with the age of weaned piglets

**Figure 3:**
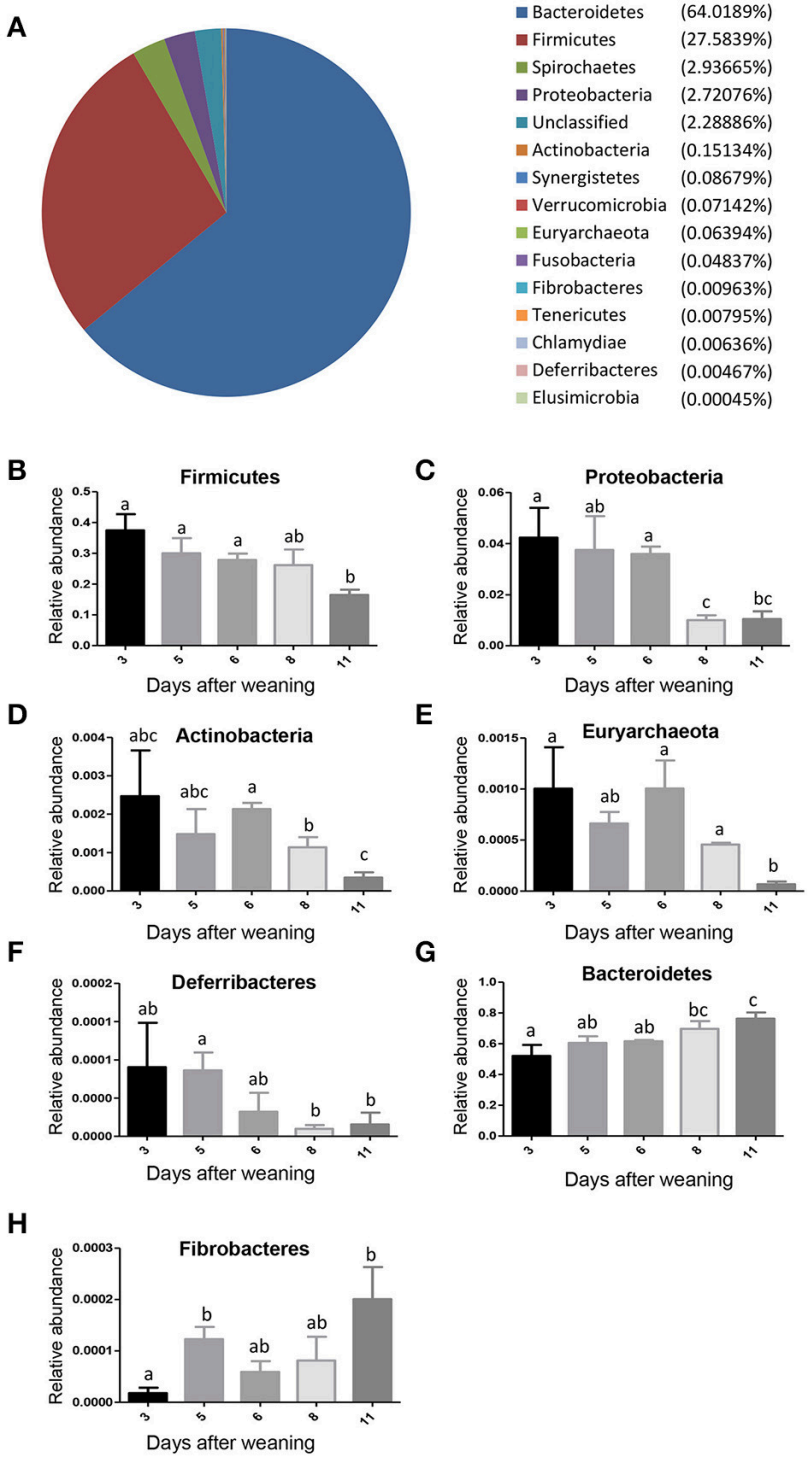
**The shifts in the gut bacterial compositions at phylum level in miniature piglets during the early period after weaning. (A)** The average proportional distributions of gut bacterial phyla identified in piglets at 5 sampled time points (3 days after weaning, 5 days after weaning, 6 days after weaning, 8 days after weaning, 11 days after weaning). **(B)** The change in the relative abundance of phylum Firmicutes with the age of piglets. **(C)** The change in the relative abundance of phylum Proteobacteria with the age of piglets. **(D)** The change in the relative abundance of phylum Actinobacteria with the age of piglets. **(E)** The change in the relative abundance of phylum Euryarchaeota with the age of piglets. **(F)** The change in the relative abundance of phylum Deferribacteres with the age of piglets. **(G)** The change in the relative abundance of phylum Bacteroidetes with the age of piglets. **(H)** The change in the relative abundance of phylum Fibrobacteres with the age of piglets. Metastats analysis was applied to identify the significantly differentially abundant phyla among groups and detailed data were presented in the Supplementary Data 1. Different letters above the bars denotes significantly differentially abundant phyla among groups.

Those alterations in the gut bacterial community diversities were accompanied by significant shifts in the gut bacterial taxonomic compositions with the age of weaned piglets. After the bacterial OTU representative sequences were taxonomically classified, the results showed 4 dominant phyla (Bacteroidetes, Firmicutes, Spirochaetes, and Proteobacteria), which consisted of over 1% of total sequences on average, were present in the bacterial communities (Figure 3A). Bacteroidetes, which consisted of ~64% of total sequences on average, was the most abundant phylum, followed by the phylum Firmicutes composed of ~28% of total sequences on average. To evaluate how the gut bacterial taxonomic compositions at phylum level altered as the piglets aged, Metastats analysis was applied to identify the differentially abundant phyla among groups. The results demonstrated significant decreases in the relative abundances of 5 phyla (Firmicutes, Proteobacteria, Actinobacteria, Euryarchaeota, and Deferribacteres) with age (Figures 3B–F). The relative abundances of 3 phyla (Tenericutes, Fusobacteria, and Synergistetes) also showed decreased trends with the age of piglets. However, the bacterial community showed significant increases in the relative abundance of only two phyla (Bacteroidetes and Fibrobacteres) as the piglets aged (Figures 3G,H).

**Figure 4:**
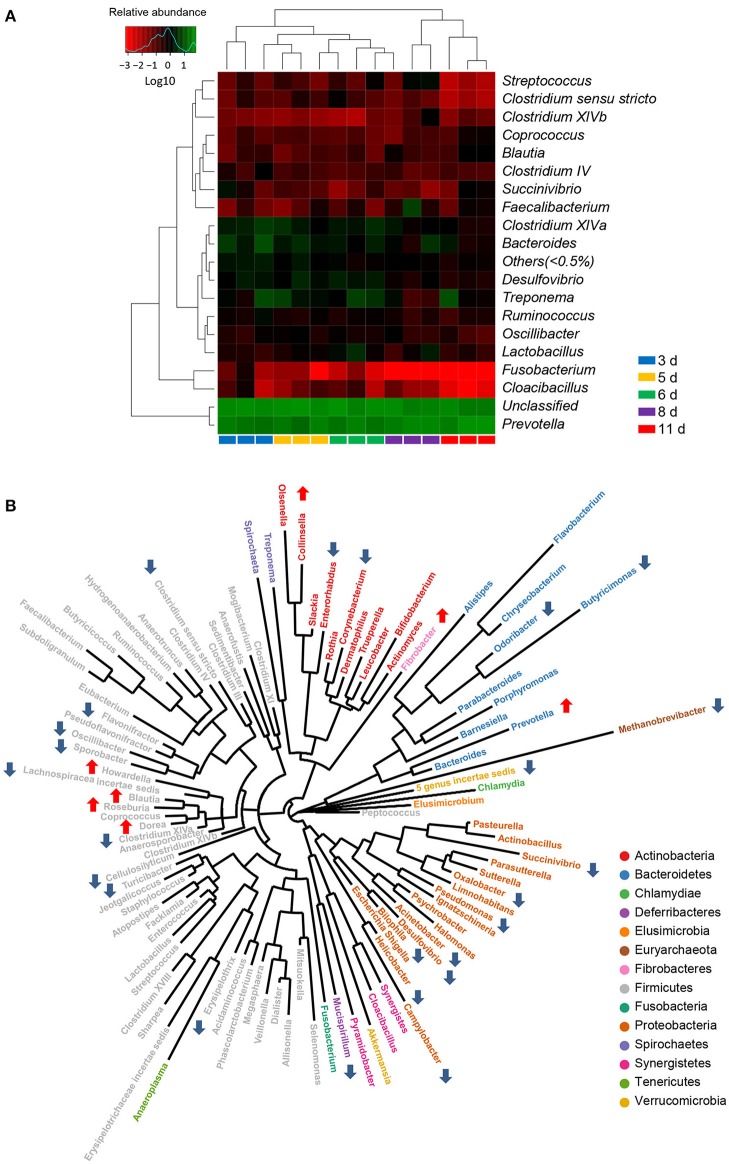
**Significant alterations in the gut bacterial compositions at genus level in miniature piglets during the early period after weaning. (A)** Heat map and hierarchical clustering of genera in the gut bacterial communities of piglets at 5 sampled time points (3 d, 3 days after weaning; 5 d, 5 days after weaning; 6 d, 6 days after weaning; 8 d, 8 days after weaning; 11 d, 11 days after weaning). The values of color in the heat map represent the normalized relative abundances of genera (Log 10). **(B)** Phylogenetic tree was constructed from the genera identified in the gut bacterial communities of weaned piglets. Up arrow indicated that the relative abundance of the corresponding genus significantly increased with the age of piglets, whereas down arrow indicated that the relative abundance of the corresponding genus significantly decreased with the age of piglets. Metastats analysis was applied to identify the significantly differentially abundant bacterial genera among groups and detailed data were presented in the Supplementary Data 2.

To further investigate the taxonomic compositions of weaned piglets, a total of 101 genera were identified from the gut bacterial communities of weaned piglets. Among these genera identified, 18 abundant genera which were defined as containing more than 0.5% of the total sequences in at least one sample were detected. The 18 abundant genera were: *Prevotella, Bacteroides, Treponema, Clostridium XlVa, Desulfovibrio, Lactobacillus, Faecalibacterium, Ruminococcus, Oscillibacter, Streptococcus, Succinivibrio, Clostridium IV, Clostridium sensu stricto, Blautia, Fusobacterium, Clostridium XlVb, Cloacibacillus*, and *Coprococcus* (Figure 4A). All the 18 abundant genera plus the unclassified genera accounted for over 97% of the total sequences in the samples, regardless of the age of piglets. Genus *Prevotella* belonged to phylum Bacteroidetes was the most abundant genera in the gut bacterial communities. The genus-level cluster analysis using heat map demonstrated a higher similarity of the samples within group than that among groups and revealed a development in bacterial genus-level compositions with the age of weaned piglets (Figure 4A).

Using Metastats analysis to compare the bacterial genus-level taxonomic compositions among groups, we found that the relative abundances of 24 genera, belonged to both abundant and less-abundant genera, significantly declined as the piglets aged (Figure 4B). A significant decrease in the relative abundance of *Methanobrevibacter*, the only genus belonged to phylum Euryarchaeota, led to a significant drop in the proportion of phylum Euryarchaeota with the age of weaned piglets. Similarly, the relative abundance of phylum Deferribacteres also declined as evidenced by the significantly decreased proportion of its only genus *Mucispirillum* with the age of weaned piglets. Interestingly, the bacterial communities also showed significant decreases in the relative abundances of all differentially abundant genera belonged to phylum Proteobacteria, thereby leading to a striking drop in the proportion of phylum Proteobacteria as the piglets aged. However, the relative abundances of 7 genera (*Fibrobacter, Collinsella, Roseburia, Prevotella, Dorea, Howardella*, and *Blautia*) significantly increased with the age of weaned piglets (Figure 4B). A significant increase in the relative abundance of *Fibrobacter*, the only genus belonged to phylum Fibrobacteres, led to a significant increase in the proportion of phylum Fibrobacteres as the piglets aged. Importantly, a dramatically significant increase from 29.5 to 52.5% in the relative abundance of genus *Prevotella*, which was the only increased and the most predominant genus within phylum Bacteroidetes, resulted in an overall significant increase in the proportion of phylum Bacteroidetes as the piglets aged. Furthermore, the relative abundances of genera *Lactobacillus* and *Clostridium XI* significantly increased and subsequently decreased, whereas the relative abundance of genus *Parasutterella* significantly decreased and subsequently increased with the age of weaned piglets.

**Figure 5:**
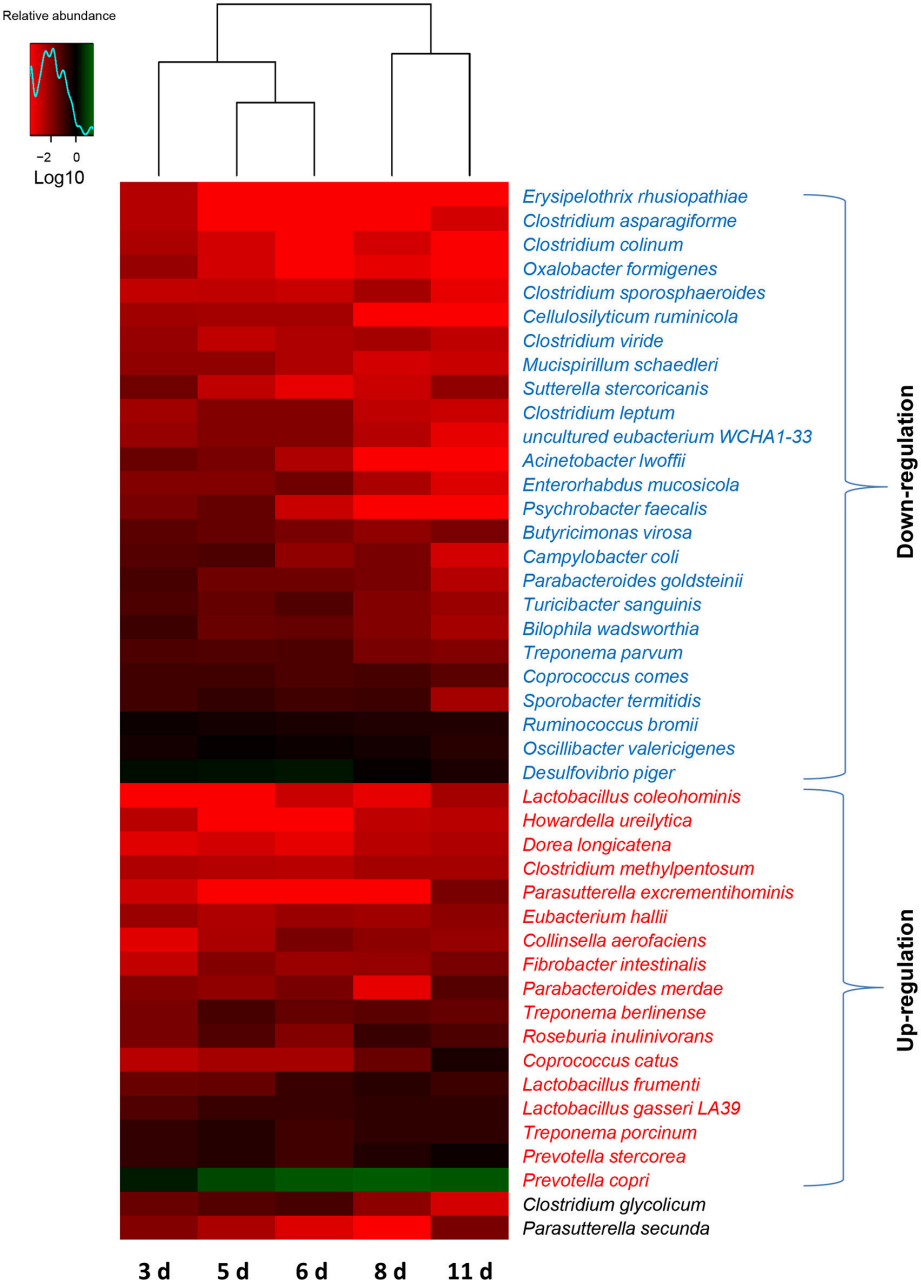
**Shifts in the gut bacterial compositions at species level in miniature piglets during the early period after weaning**. Heat map and hierarchical clustering of differentially abundant gut bacterial species in piglets at 5 sampled time points (3 d, 3 days after weaning; 5 d, 5 days after weaning; 6 d, 6 days after weaning; 8 d, 8 days after weaning; 11 d, 11 days after weaning). The values of color in the heat map represent the normalized relative abundances of species (Log 10). Detailed data for heat map were shown in the Supplementary Data 3. Metastats analysis was applied to identify the significantly differentially abundant bacterial species among groups and detailed data were presented in the Supplementary Data 4.

To further dissect the shifts of the taxonomic composition of gut microbiota with the age of weaned piglets, a total of 148 species were identified in the bacterial populations. The most abundant species was *Prevotella copri*, consisting of over 14% of the total sequences in the samples on average. There was no significant change in the relative abundances of 104 species with the age of piglets. However, the relative abundances of 25 bacterial species decreased as the piglets aged shown in Figure 5. Among them, 6 bacterial species (*Erysipelothrix rhusiopathiae, Clostridium colinum, Oxalobacter formigenes, Cellulosilyticum ruminicola, Acinetobacter lwoffii*, and *Psychrobacter faecalis*) even cannot be detected in the gut bacterial communities of piglets (11 days after weaning). However, this study demonstrated an increase in the relative abundances of 17 bacterial species with the age of piglets (Figure 5). Among them, 5 bacterial species (*Prevotella copri, Lactobacillus frumenti, Prevotella stercorea, Eubacterium hallii*, and *Treponema porcinum*) belonged to the core species which can be identified in all the samples. Importantly, among these bacterial species whose relative abundances increased as the piglets aged, 4 species (*Lactobacillus coleohominis, Lactobacillus frumenti, E. hallii*, and *Lactobacillus gasseri LA39*) can produce the antimicrobial substances, such as lactic acid, butyrate, and antimicrobial peptide. In addition, the relative abundance of species *Clostridium glycolicum* significantly increased and subsequently decreased as the piglets aged. The relative abundance of species *Parasutterella secunda* significantly decreased and subsequently increased as the piglets aged.

### Shifts in gut fungal taxonomic compositions with the age of weaned piglets

**Figure 6:**
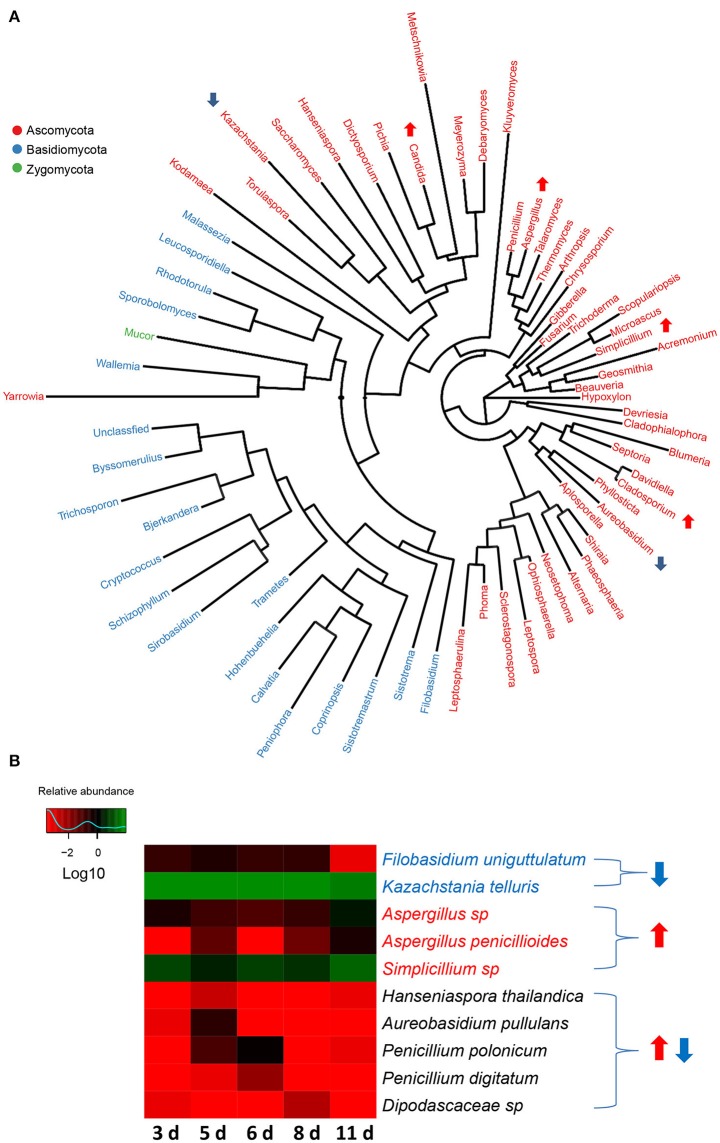
**The changes in gut fungal compositions at genus level and species level in miniature piglets during the early period after weaning. (A)** Phylogenetic tree was constructed from the genera identified in the gut fungal communities of weaned piglets. Up arrow indicated that the relative abundance of the corresponding genus significantly increased with the age of piglets, whereas down arrow indicated that the relative abundance of the corresponding genus significantly decreased with the age of piglets. Metastats analysis was applied to identify the differentially abundant fungal genera among groups and detailed data were shown in the Supplementary Data 5. **(B)** Heat map of differentially abundant gut bacterial species in piglets at 5 sampled time points (3 d, 3 days after weaning; 5 d, 5 days after weaning; 6 d, 6 days after weaning; 8 d, 8 days after weaning; 11 d, 11 days after weaning). The values of color in the heat map represent the normalized relative abundances of species (Log 10). Detailed data for the heat map were shown in the Supplementary Data 6. Metastats analysis was applied to identify the significantly differentially abundant fungal species among groups and detailed data were shown in the Supplementary Data 7.

There were 3 phyla (Zygomycota, Basidiomycota, and Ascomycota) identified in the fungal communities of weaned piglets using RDP classifier. Phylum Ascomycota, which accounted for more than 97% of total sequences in the samples, regardless of age, was the most dominant phylum. Unlike the gut bacterial communities, gut fungal communities showed no significant alteration in the proportions of phyla with the age of piglets, further supporting the relatively stabilized gut fungal communities in piglets during the early period after weaning. A total of 67 genera were detected in fungal communities of weaned piglets (Figure 6A). Among them, *Kazachstania*, a member of phylum Ascomycota, was the major genus accounting for over 78% of total sequences in the fungal communities on average. After analyzing the data using Metastats, we found that the relative abundances of 4 genera (*Aspergillus, Cladosporium, Simplicillium*, and *Candida*) significantly increased with the age of piglets (Figure 6A). However, the relative abundances of 2 genera (*Kazachstania* and *Aureobasidium*) significantly decreased with the age of piglets (Figure 6A). In addition, the relative abundances of 2 genera (*Hanseniaspora* and *Penicillium*) significantly increased and subsequently decreased with the age of piglets. These differentially abundant genera in fungal communities all belonged to the phylum Ascomycota, indicating the relatively stabilized fungal taxonomic compositions for phyla Zygomycota and Basidiomycota as the piglets aged (Figure 6A).

At species level, altogether 124 species were identified in the fungal communities of weaned piglets. Unlike the gut bacterial communities, all the sequences can be annotated at species level. The most abundant fungal species was *Kazachstania telluris*, belonged to genus *Kazachstania*, consisting of over 78% of the total sequences in the samples on average. Metastat analysis indicated that gut fungal communities showed significant decreases in the relative abundances of 2 species (*Filobasidium uniguttulatum* and *K. telluris*) and significant increases in the relative abundance of 3 species (*Aspergillus* sp., *Aspergillus penicillioides*, and *Simplicillium* sp.) with the age of piglets (Figure 6B). In addition, the relative abundance of 5 species (*Hanseniaspora thailandica, Aureobasidium pullulans, Penicillium polonicum, Penicillium digitatum*, and *Dipodascaceae* sp) significantly increased and subsequently decreased as the piglets aged (Figure 6B).

### Functional maturation of the gut bacterial community with the age of weaned piglets

**Figure 7:**
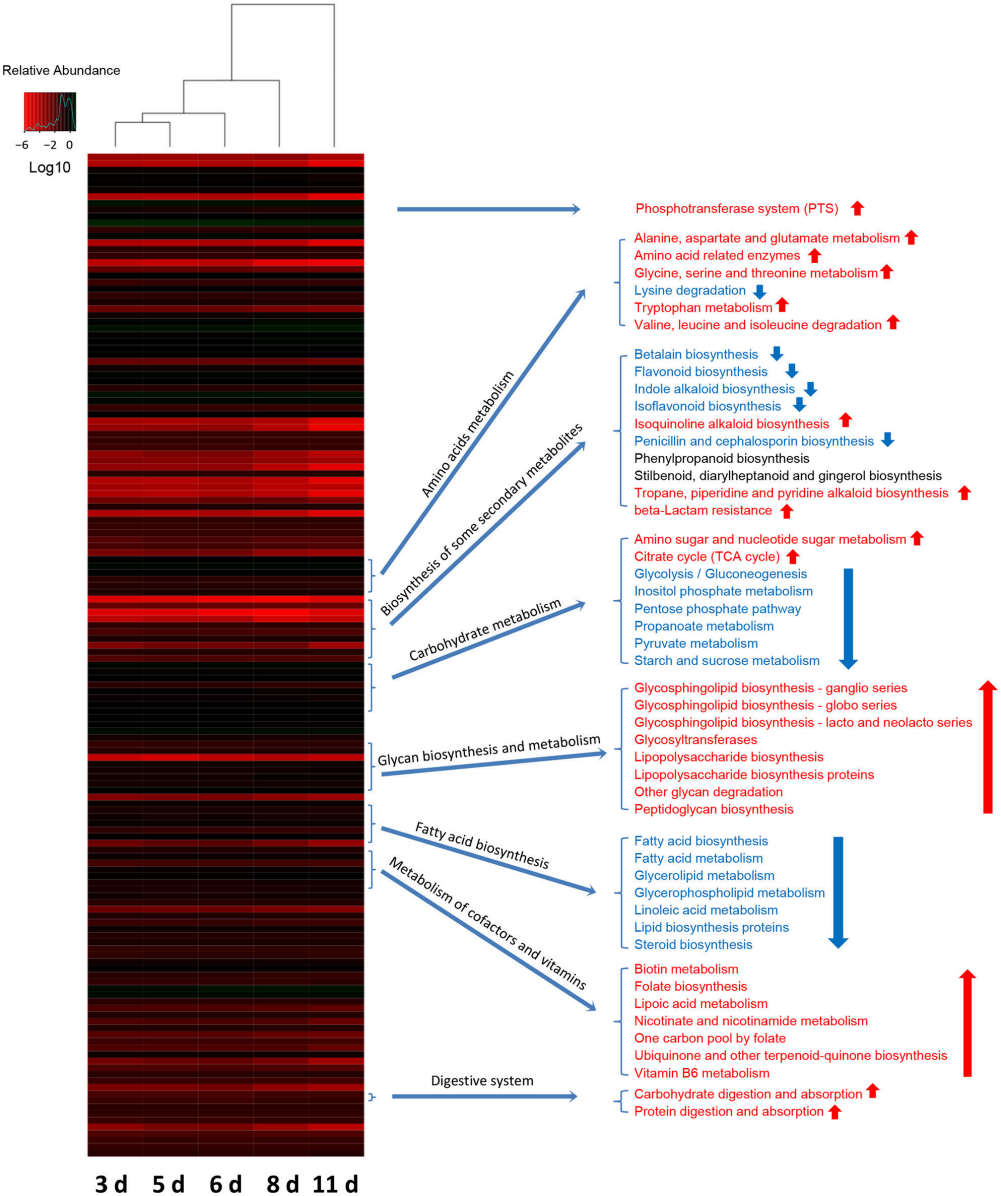
**Shifts in gut bacterial functional profiles as the miniature piglets aged**. Heat map and hierarchical clustering of differentially abundant KEGG pathways identified at 5 sampled time points (3 d, 3 days after weaning; 5 d, 5 days after weaning; 6 d, 6 days after weaning; 8 d, 8 days after weaning; 11 d, 11 days after weaning). The values of color in the heat map represent the normalized relative abundance of KEGG pathways (Log 10). Detailed data for the heat map were shown in the Supplementary Data 8. Metastats analysis was applied to identify the significantly differentially abundant KEGG pathways among groups and detailed data were shown in the Supplementary Data 9.

To investigate how the functional capacity of the intestinal bacterial community developed during the early period after weaning in piglets, PICRUSt approach was used to analyze the KEGG pathways compositions in bacterial populations. The PICRUSt analyses suggested the distinct nutrient source utilization of the gut bacteria in weaned piglets at 5 sampled time points (Figure 7). Phosphotransferase system (PTS) genes required for carbohydrate uptake were the most abundant in the gut bacterial communities of piglets (3 days after weaning), possibly due to the sudden intestinal malnutrition caused by underfeeding after weaning. In contrast, the results showed a significant increased digestive system with the age of piglets as evidenced by the increased proportions of the genes for carbohydrate digestion and absorption and protein digestion and absorption. In particular, the relative abundances of the genes involved in amino sugar and nucleotide sugar metabolism and other glycan degradation significantly increased with the age of piglets, suggesting an enhancement of bacterial complex carbohydrate metabolism capacity as the piglets aged. The bacterial community also showed a shift in the carbohydrate metabolism as the piglets grow older. The proportions of the genes for glycolysis/gluconeogenesis, inositol phosphate metabolism, pentose phosphate pathway, propanoate metabolism, pyruvate metabolism, and starch and sucrose metabolism significantly decreased, whereas the proportions of the genes involved in citrate cycle (TCA cycle) significantly increased with the age of piglets. The amino acids metabolisms in gut bacterial communities also varied as the piglets aged. The bacterial community showed significantly increased relative abundances of the genes for alanine, aspartate and glutamate metabolism, amino acid related enzymes, glycine, serine and threonine metabolism, tryptophan metabolism and valine, leucine and isoleucine biosynthesis, whereas a significantly decreased relative abundance of the genes for lysine degradation. Furthermore, the bacterial lipid metabolisms were significantly reduced with the age of piglets as evidenced by the decreased proportions of genes for fatty acid biosynthesis, fatty acid metabolism, glycerolipid metabolism, glycerophospholipid metabolism, linoleic acid metabolism, lipid biosynthesis proteins, and steroid biosynthesis.

The biosynthesis of vitamin B is critical for the bioconversion from nutrients into energy. In the present study, the relative abundances of genes required for biotin (vitamin B7) metabolism and pyridoxal (vitamin B6) metabolism both significantly increased with the age of piglets (Figure 7). The genes for the biosynthesis of folate (vitamin B9), an essential B vitamins also involved in DNA synthesis and repair, showed a significantly increased relative abundance as the piglets aged (Figure 7). As the piglets grow order, the relative abundances of genes for metabolism of cofactors, such as lipoic acid metabolism, nicotinate, and nicotinamide metabolism, one carbon pool by folate, ubiquinone and other terpenoid-quinone biosynthesis significantly increased (Figure 7). Furthermore, the results showed significant increases in the proportions of genes required for glycan biosynthesis and metabolisms, such as glycosphingolipid biosynthesis-ganglio series, glycosphingolipid biosynthesis-globo series, Glycosphingolipid biosynthesis-lacto, and neolacto series, glycosyltransferases, lipopolysaccharide biosynthesis, lipopolysaccharide biosynthesis aproteins, other glycan degradation, and peptidoglycan biosynthesis (Figure 7). Notably, the Bacterial biosynthesis of some secondary metabolites varied with age. The relative abundances of genes for isoquinoline alkaloid biosynthesis, tropane, piperidine, and pyridine alkaloid biosynthesis, and beta-Lactam resistance significantly increased with the age of piglets. However, the relative abundances of genes involved in betalain biosynthesis, flavonoid biosynthesis, indole alkaloid biosynthesis, isoflavonoid biosynthesis, and penicillin and cephalosporin biosynthesis significantly decreased as the piglets aged (Figure 7).

## Discussion

This study investigated the gut microbial shifts in Congjiang miniature piglets during the early period after weaning. The results revealed the developments of gut microbiota compositions and functional maturation of gut bacterial communities in the Congjiang miniature piglets during the early period after weaning.

The present study showed a significantly decreased alpha diversity in the gut bacterial community with the age of piglets. However, recent studies have demonstrated a significantly increased gut bacterial alpha diversity with age at ~1-month intervals after weaning in pigs (Niu et al., 2015; Zhao et al., 2015). It seems likely that gut bacterial community undergoes an increased alpha diversity from weaning to adulthood in pigs on the whole, whereas shows a decreased alpha diversity during the early period after weaning. Growing evidences have linked gut microbial alterations to diets (Maslowski and Mackay, 2011; Doré and Blottière, 2015). So it is possible that the significantly decreased alpha diversity in the gut bacterial community with age during the early period after weaning may be the result of sudden diet transition from breast milk to solid feed after weaning in piglets.

Consistent with previous studies on pigs (Kim et al., 2012; Looft et al., 2012), this study demonstrated that Bacteroidetes and Firmicutes were the two most dominant phyla in gut bacterial communities of miniature piglets. The results obtained in those studies based on human infants indicated that Bacteroidetes and Firmicutes were the most prevalent phyla, followed by Actinobacteria and Proteobacteria (Backhed et al., 2015; Kostic et al., 2015), suggesting the similarities between miniature piglets' gut bacterial taxonomic compositions and human infants' gut bacterial taxonomic compositions. The results of this study also showed that genus *Prevotella* belonged to phylum Bacteroidetes was the most abundant genera in gut bacterial communities, as was shown to be a feature of the gut microbiota in pigs (Lamendella et al., 2011; Kim et al., 2012; Looft et al., 2012). Our results demonstrated significant declines in the relative abundances of 5 phyla (Firmicutes, Proteobacteria, Actinobacteria, Euryarchaeota, and Deferribacteres) and significant increases in the relative abundances of 2 phyla (Bacteroidetes and Fibrobacteres) with the age of the miniature piglets. However, an earlier study indicated that the relative abundances of 3 phyla (Fusobacteria, Lentisphaerae, and Synergistetes) significantly decreased and 2 phyla (Tenericutes and TM7) significantly increased as the pig aged (Niu et al., 2015). The reason of the distinct shifts with age at phylum level could be that our studies focused on the gut microbiota during the early period after weaning in piglets, whereas their studies investigated the gut microbiota during the period from weaning to adulthood in pigs. At genus level, the bacterial communities showed that the relative abundances of 7 genera (*Fibrobacter, Collinsella, Roseburia, Prevotella, Dorea, Howardella*, and *Blautia*) significantly increased with the age of weaned miniature piglets. Among them, 4 genera (*Roseburia, Prevotella, Dorea*, and *Blautia*) were also demonstrated increased relative abundances with the age of infants (from newborn to 12 months) in a recent study based on the gut microbiota of human infants (Backhed et al., 2015). Our results also indicated that the relative abundances of genera *Lactobacillus* significantly increased and subsequently decreased with the age of piglets, which is also in line with the results of study based on the gut microbiota of infants (Backhed et al., 2015).

Gut microbial shifts at species level have absorbed a mass of attentions due to that gut microbiota can be modified for therapeutic applications (Buffie et al., 2015; Schieber et al., 2015; Sivan et al., 2015; Vétizou et al., 2015). In the present study, 6 bacterial species (*Erysipelothrix rhusiopathiae, Clostridium colinum, Oxalobacter formigenes, Cellulosilyticum ruminicola, Acinetobacter lwoffii*, and *Psychrobacter faecalis*) even cannot be detected in the gut bacterial communities of piglets (11 days after weaning), suggesting that these bacterial species may could not adapt to the intestinal tract environment with the age of weaned miniature piglets. However, among those species whose relative abundances increased as the piglets aged, 5 bacterial species (*Prevotella copri, Lactobacillus frumenti, Prevotella stercorea, E. hallii*, and *Treponema porcinum*) can be detected in all samples, suggesting that these species could adapt to the gut environment in miniature piglets well and may have benefits for host health. Furthermore, among these bacterial species whose relative abundances increased as the piglets aged, 4 species (*L. coleohominis, E. hallii, Lactobacillus frumenti*, and *Lactobacillus gasseri LA39*) can produce antimicrobial substances, such as lactic acid, butyrate, and antimicrobial peptide. It was widely recognized that these antimicrobial substances contribute to the intestinal mucosal barrier maintenance. Thus, these 4 bacterial species may be the candidates for probiotics applied in weaned piglets or human infants.

Together with the development of gut microbiota during the early period after weaning in miniature piglets, the functional maturation of the microbiome was also assessed using PICRUSt. PICRUSt, making up for the shortage of 16S rDNA gene studies, has been an effective tool to predict the functional profiles of bacterial communities (Langille et al., 2013; Buffie et al., 2015). Recently, Langille et al. have demonstrated that human-associated microbiota samples had a mean NSTI value (0.03 ± 0.2 *s.d.*), other mammalian-associated microbiota samples had a mean NSTI value (0.14 ± 0.06 *s.d.*), and soil had a mean NSTI value (0.17 ± 0.02 *s.d.*). Thus, our piglets fecal samples, which had a mean NTS1 values (0.1469 ± 0.01902 *s.d.*) showed an ideal accuracy of PICRUSt prediction. Consistent with a previous study based on the gut microbiome in infants (Backhed et al., 2015), the results of this study suggested an enhancement of carbohydrate digestion and absorption capacity, especially the complex carbohydrate metabolism capacity, in the gut microbiome with the age of piglets. Furthermore, the protein digestion and absorption capacity of gut microbiome also significantly enhanced with the age of piglets in this study. The significantly increased relative abundances in almost all the KEGG pathways (except the lysine degradation) belonged to amino acids metabolisms, further supporting the enhanced capacity of protein digestion and absorption as the piglets aged. Considering that the gut microbes utilize the nutrients in host intestinal tract for survival, so it is possible that the enhancement of gut bacterial digestive system in carbohydrate and protein is the result of the increased intake of solid feed composed of more complex carbohydrates and proteins than whose in sow's milk as the piglets aged. Our results also suggested that the bacterial capacity for the metabolism of vitamin B also significantly increased with the age of piglets, which is in line with the results obtained in those earlier studies based on human infants (Yatsunenko et al., 2012; Backhed et al., 2015). The intestinal microbiota is a key producer of vitamins which play an important role in host health, implying the importance of increased gut bacterial vitamin B metabolism as the piglets aged. There was a striking increase in the glycan biosynthesis and metabolism capacity of the gut microbiome as a function of age. Given the lipopolysaccharide and peptidoglycan biosynthesis is vital for the bacterial cell wall and membrane biosynthesis, it could be assured that the growth and proliferation rates of the gut bacteria could increased as the piglets aged.

To our knowledge, extremely few studies have investigated the gut fungal communities of pigs. In the present study, we used ITS2 high-throughput sequencing, a culture-independent method, to identify the gut fungal communities. The results obtained provided an insight into fungal communities in weaned piglets. This study showed that *Kazachstania* was the most predominant genus accounting for over 78% of total sequences in the fungal communities on average. However, previous studies on the mice (Iliev et al., 2012; Dollive et al., 2013) and white pine beetle (Hu et al., 2015), an insect, demonstrated that *Candida* was the most abundant genus in the gut fungal communities, which was inconsistent with the results of our studies. In addition, there is no common abundant gut fungal genera between miniature piglets and white pine beetles, whereas only 3 abundant gut genera (*Aspergillus, Alternaria*, and *Trichosporon*) were identified in both miniature piglets and mice. These differences suggested a specificity of gut fungal compositions in miniature piglets, compared to that in mice and insect.

In sum, the present study revealed the development of both gut bacterial and fungal communities with the age of weaned piglets. This study also suggested the functional maturations of gut bacterial communities characterized by increased digestive system, glycan biosynthesis and metabolism, and vitamin B biosynthesis. The results of this study suggested the similarities between miniature piglets' gut microbiota and human infants' gut microbiota according to those previous studies based on human infants. Thus, our study may facilitate the development of animal model for research on the infant gut microbiota.

## Author contributions

The authors' contributions are as follows: JH, YN, and XY designed the research. JH, YN, YZ, ZW, and QF conducted the research. JH, YN, and JC analyzed the data. JH, YN, and XY wrote the manuscript. All authors read and approved the final version of the manuscript.

## Funding

This work was supported by the National Natural Science Foundation of China (31322053, 31520103915, and 31172290), the National Key Basic Research Program of China (973 Program) (2013CB127305), New Century Excellent Talents in University (NCET-12-0860), the Hubei Province Distinguished Young Scholar (2012FFA015), and the Fundamental Research Funds for the Central Universities (2013PY056, 2662015PY111, and 2013JQ001).

### Conflict of interest statement

The authors declare that the research was conducted in the absence of any commercial or financial relationships that could be construed as a potential conflict of interest.
