# Supplementary material for: Gradual Changes of Gut Microbiota in Weaned Miniature Piglets
Source: Front Microbiol. 2016 Nov 2;7:1727. doi: 10.3389/fmicb.2016.01727 (PMC5090779; doi:10.3389/fmicb.2016.01727)
Supplement: Supplementary file 1 [file Presentation1.PDF]

Gradual Changes of Gut Microbiota in Weaned Miniature Piglets

Supplementary materials legends

Supplementary Figure 1 | Experiment design. Congjiang miniature piglets were weaned at the age of 21 days. This study amplicon-sequenced fecal samples from Congjiang miniature piglets at 5 sampled time points (3, 5, 6, 8, and 11 days) after weaning.

Supplementary Data 1 Detailed data for Metastats analysis of the gut bacterial phyla.

Supplementary Data 2 Detailed data for Metastats analysis of the gut bacterial genera.

Supplementary Data 3 Detailed data for the heat map showing the differentially abundant gut bacterial species.

Supplementary Data 4 Detailed data for Metastats analysis of the gut bacterial species.

Supplementary Data 5 Detailed data for Metastats analysis of the gut fungal genera.

Supplementary Data 6 Detailed data for the heat map showing the differentially abundant gut fungal species.

Supplementary Data 7 Detailed data for Metastats analysis of the gut fungal species.

Supplementary Data 8 Detailed data for the heat map showing the differentially abundant KEGG pathways.

Supplementary Data 9 Detailed data for STAMP analysis of the KEGG pathways.
