# Supplementary figures and images for: Gradual Changes of Gut Microbiota in Weaned Miniature Piglets
Source: Front Microbiol. 2016 Nov 2;7:1727. doi: 10.3389/fmicb.2016.01727 (PMC5090779; doi:10.3389/fmicb.2016.01727)

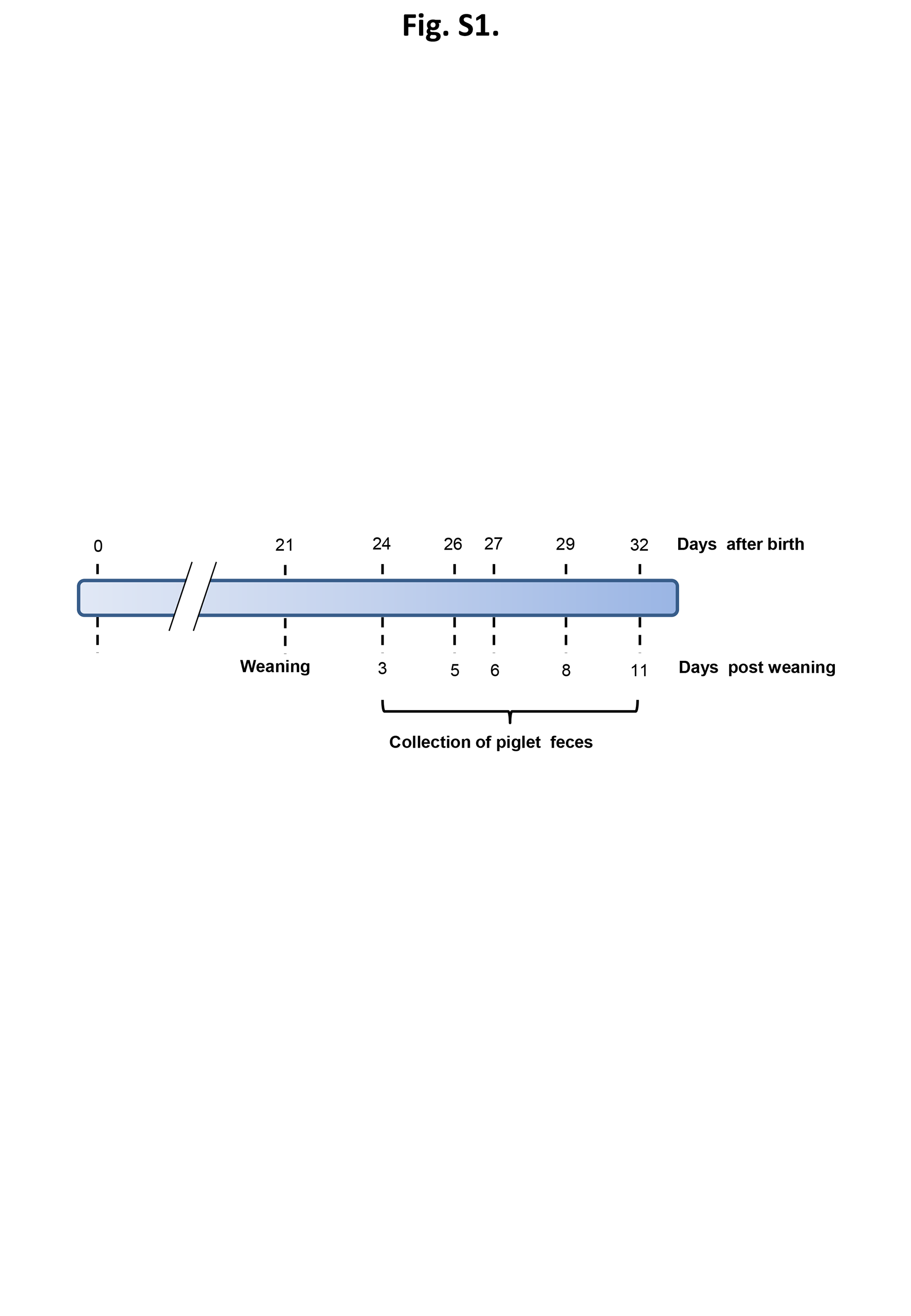

Supplement: Supplementary file 11 [file Image1.TIF]
